# Geometric‐Mean Fitness Does Not Correspond to Long‐Term Survival Probability

**DOI:** 10.1002/ece3.72809

**Published:** 2025-12-28

**Authors:** Takuya Okabe, Jin Yoshimura, Hiromu Ito

**Affiliations:** ^1^ Graduate School of Integrated Science and Technology Shizuoka University Hamamatsu Japan; ^2^ Department of International Health and Medical Anthropology, Institute of Tropical Medicine Nagasaki University Nagasaki Japan; ^3^ Marine Biosystems Research Center Chiba University Chiba Japan; ^4^ Department of Biological Science Tokyo Metropolitan University Tokyo Japan

## Abstract

Unpredictable randomness plays a crucial role in long‐term sustainability of biological systems. The population growth of a species in variable environments is typically described in terms of a long‐term measure like geometric‐mean fitness, or geometric mean of stochastic growth rates. However, quantitative understanding of the relationship between these fitness measures and long‐term survival probability remains a critical, and often overlooked, aspect of ecological modeling. Here, we investigate this relationship using large‐scale numerical simulations, focusing on the implications for bet‐hedging strategies. To this end, we develop two individual‐based growth models incorporating randomly varying growth rates. Our simulations reveal that a one‐to‐one correspondence, or monotonic relationship, does not exist between geometric mean fitness and survival probability. Specifically, higher geometric mean fitness does not necessarily correlate with increased survival probability. These findings challenge the assumption of a universal, time‐independent measure of long‐term fitness and suggest that the “optimal” survival strategy is likely contingent on the timescale of observation.

## Graphical abstract

This study investigates the relationship between geometric mean fitness and survival probability in variable environments, using simulations to explore bet‐hedging strategies. Results reveal no direct correlation between these measures, challenging the assumption of a universal fitness metric and highlighting the importance of timescale in determining optimal survival strategies.

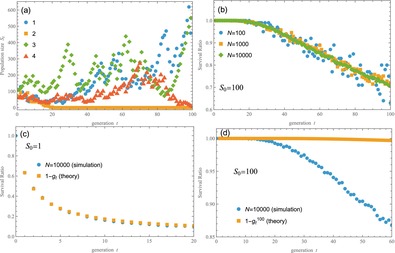

## 1 Introduction

The long‐term fate of a species' population is significantly influenced by various random factors, such as environmental changes and natural disasters, which can unpredictably alter survival and reproduction rates. Indeed, randomness may cause the extinction of a population that would be predicted to persist indefinitely if it were not for randomness and/or if its effect is simplistically considered as an average, so much so that random fluctuations in population size are of fundamental importance to ecology and conservation biology. Species have evolved to adapt to regular and irregular environmental fluctuations. Long‐term persistence of a population under variable environments may be threatened because of random variation in fitness, or growth rate. The geometric‐mean fitness (growth rate) has been customarily considered as a long‐term index with which to measure long‐term performance of a population under variable environments (Cohen 1966; Seger and Brockmann 1987; Philippi and Seger 1989; Yoshimura and Clark 1991). Its usefulness has been widely recognized especially in understanding biological bet‐hedging problems (Haaland et al. 2019; Yasui 2001, 2022). The concept of geometric‐mean fitness has been regarded as a metric for assessing long‐term outcomes, specifically in relation to the long‐term survival probability of populations experiencing variable environmental conditions. This application of the geometric‐mean fitness concept dates back to its use in addressing seed bank problems (Cohen 1966). Delayed seed germination decreases the annual average number of seeds that sprout, but enhances their chances of long‐term survival as reflected by the geometric mean of fitness values across generations. Geometric‐mean fitness is a critical tool in evolutionary biology because it provides a more biologically meaningful way to track changes in population fitness than a simple arithmetic mean. Despite its significance, however, there remains an ongoing need for deeper quantitative insights into how geometric‐mean fitness correlates with the long‐term survival probabilities of populations. This area continues to be a pivotal yet contentious topic within ecological modeling, necessitating further exploration and clarification.

Population growth is modeled with a fluctuating growth rate, varying between $l_{1}$ and $l_{2}$ with probabilities $p_{1}$ and $p_{2}\left(=1-p_{1}\right)$. The geometric mean of these growth rates, $G=l_{1}^{p_{1}}l_{2}^{p_{2}}$, represents the long‐term average population growth. On the other hand, extinction probability is defined by the proportion of populations that would become extinct under randomly varying environmental conditions. The survival ratio is then calculated as one minus the extinction probability. Extinction probability is defined unequivocally only when the discreteness of population numbers is considered; since growth rates $l_{1}$ and $l_{2}$ are positive, the population size varying with these rates will not reach zero, though it can approach arbitrarily close to extinction. The number of individuals varies randomly even if the average growth rate is held constant because the individual number can only take a whole number (0, 1, 2, etc.). Extinction is the reduction of population size from some positive number to zero as a result of random variation in individual number. Even if the sample average changes smoothly (not randomly), individual numbers can still fluctuate unpredictably. The kind of randomness in individual variations, known as demographic stochasticity (Sæther and Engen 2015), is particularly significant for populations with small sizes (Haccou and Iwasa 1996; Engen et al. 1998; Athreya et al. 2004). Thus, numerical evaluation of survival probability necessitates the use of discrete models that generalize continuous growth models, such that population size at each generation takes a whole number (integer) while respecting that, on average, the size agrees with the prediction of the continuous model. This generalization can be implemented in several different ways. We showcase two new implementations detailed below.

This study investigates the relationship between geometric‐mean fitness and survival probability through numerical simulations. We focus on the formal aspects of this relationship, rather than specific biological problems. Our primary contribution is to demonstrate, using simplified models and freely varied parameters, that the correspondence is not always straightforward. In the next section, we introduce two new models differing in the probabilistic assignment of individual offspring numbers. In the Methods section, we explain the simulation procedure, followed by the presentation of results from numerical simulations for various cases in the Results section. The final section provides discussions of the implications of these findings. Since we are interested in the formal aspects, our analysis concentrate on simple models with a limited set of parameters due to computational constraints in calculation time and memory usage.

## 2 Models

**FIGURE 1:**
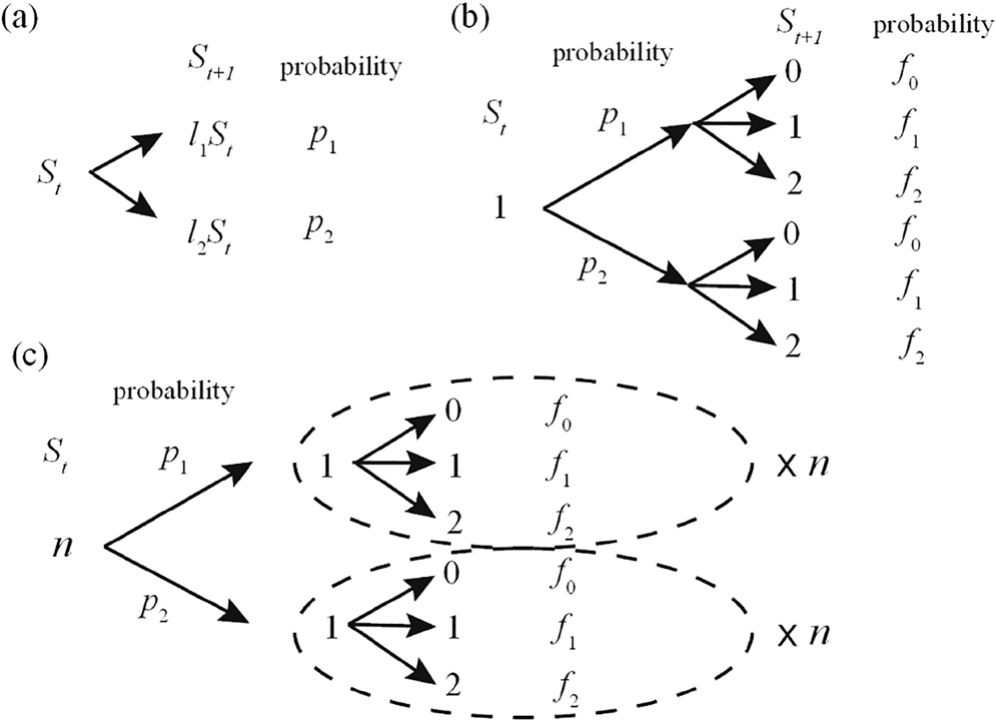
Graphical explanation of models. (a) In the continuous model in Equation (1), population size $S_{t+1}$ equals either (i) $l_{1}$ times $S_{t}$ with probability $p_{1}$ or (ii) $l_{2}$ times $S_{t}$ with probability $p_{2}\left(=1-p_{1}\right)$. The size $S_{t}$ takes values among non‐negative real numbers. (b) In Model 1, $S_{t}$ is restricted to non‐negative integer values. In Model 2, $S_{t}$ is limited to 0, 1 and 2. When $S_{t}=1$, the probability that $S_{t+1}$ takes an integer k is denoted as $f_{k}$. (c) For $S_{t}=n$, $S_{t+1}$ is obtained by repeating the process (b) n times. The probabilities $f_{k}$ are selected to ensure that the discrete processes described in (b) and (c) effectively replicate the behavior of the continuous model in (a) when considered on average. This alignment ensures consistency between the continuous model (a) and its discrete counterparts (b) and (c).

We examine a base model in which the growth rate of a population varies between $l_{1}$ and $l_{2}$, occurring with probabilities $p_{1}$ and $p_{2}=\left(1-p_{1}\right)$, respectively (Figure 1a). This is a Malthusian growth model with random growth rates (Lewontin and Cohen 1969). At every generation (discrete time) t, population size $S_{t}$ changes by a growth rate l, i.e.,

(1) $$S_{t+1}={\mathrm{lS}}_{t},$$

where l takes either one of two positive values $l_{1}$ and $l_{2}$ with probabilities $p_{1}$ and $p_{2}=1-p_{1}$, respectively. For example, the two values correspond to the growth rates in good and bad environmental conditions. We assume an asexual population with non‐overlapping generations.

The expected value (arithmetic mean) of the growth rates is given by

(2) $$E=p_{1}l_{1}+p_{2}l_{2}.$$

The geometric mean of the growth rates is given by

(3) $$G=l_{1}^{p_{1}}l_{2}^{p_{2}}=e^{\mu_{\log l}},$$

where $\mu_{\log l}$ is the expected value of the logarithmic growth rates,

(4) $$\mu_{\log l}=p_{1}\log l_{1}+p_{2}\log l_{2}.$$

In the Results section, we conduct numerical simulations by varying $l_{1},l_{2}$ and $p_{1}$ while keeping E or G constant. This is due to our interest in the parameter dependence of survival probability. In addition to the mean $\mu_{\log l}$, the variance of the logarithmic growth rates,

(5) $$\sigma_{\log l}^{2}=\sum_{i}p_{i}{\left(\log l_{i}-\mu_{\log l}\right)}^{2},$$

has also been highlighted as a significant factor in understanding the variability of growth rates. While the ultimate fate of population growth or decline corresponds to whether G is larger or smaller than 1, the ‘extinction’ probability, the probability that the population size will fall below a small threshold value, depends not only on G but also on the variance $\sigma_{\log l}^{2}$ (Tuljapurkar and Orzack 1980; Tuljapurkar et al. 2009; Trotter et al. 2013; Okabe and Yoshimura 2022; Zhou and Xue 2022; Cavallero et al. 2025). For the sake of comparison with the geometric mean G, we consider the modified geometric mean (Okabe and Yoshimura 2022; Cavallero et al. 2025),

(6) $$G^{\prime }=e^{\mu_{\log l}/\sigma_{\log l}}.$$

While the population size, $S_{t}$, can assume any positive value in Equation (1), we introduce restrictions to ensure $S_{t}$ takes only integer values (0, 1, 2, etc.).

### 2.1 Model 1

The first model posits that each of $S_{t}$ individuals gives birth to a random number of offspring, distributed according to a Poisson distribution with mean value l. The probability of an individual having k offspring is given by $f_{k}=l_{i}^{k}e^{-l_{i}}/k!$, where $l_{i}$ is either $l_{1}$ or $l_{2}$ (Figure 1b). This distribution is characterized by the properties that the probabilities sum to unity $\left(\sum_{k=0}^{\infty }f_{k}=1\right)$ and the expected number of offspring equals $l_{i}$ $\left(\sum_{k=0}^{\infty }{\mathrm{kf}}_{k}=l_{i}\right)$. Consequently, this model is consistent with the original model in Equation (1) when considering the statistical average. For $S_{t}=n$ individuals, this process is repeated n times independently (Figure 1c).

If all $S_{t}$ individuals happen to give zero offspring, the population goes extinct at the next generation ($S_{t+1}=0$). This occurs with probability $f_{0}^{S_{t}}=e^{-l_{i}S_{t}}$. Thus, the larger the growth rate $l_{i}$, the smaller the extinction probability, and the larger the survival probability. While this appears plausible, this correlation stems from the assumption of the Poisson distribution, which may or may not be appropriate in reality. Ecological systems often present a trade‐off between maximizing the arithmetic‐mean growth rate and the geometric‐mean growth rate (Philippi and Seger 1989; Yoshimura and Jansen 1996). Since the trade‐off occurs when the above correlation does not hold true, we consider another model free from this implicit correlation.

### 2.2 Model 2

In the second model, each individual produces either zero, one or two offspring, with the probabilities of each outcome being $f_{0}$, $f_{1}$ and $f_{2}$, respectively. The expected value of offspring, $f_{1}+2f_{2}$, is equated with the growth rate $l_{i}$ $\left(i=1,2\right)$ (Figure 1a). Since the probabilities add up to unity, $f_{0}+f_{1}+f_{2}=1$, we obtain $f_{1}=2\left(1-f_{0}\right)-l_{i}$ and $f_{2}=f_{0}+l_{i}-1$ (Figure 1b). Unlike the first model, the probability of extinction $S_{t+1}=0$ from the last individual $S_{t}=1$ takes a constant value $f_{0}$, independent of $l_{i}$. Accordingly, the smaller the growth rate $l_{i}$, the larger the probability $f_{1}$, and the smaller the probability $f_{2}$. Beyond its low computational cost, a key benefit of this model is its ability to consider two distinct extinction factors separately: the probability of a population reaching a minimum size ($S_{t}=1$), and the probability that the last individual will fail to reproduce ($S_{t+1}=0$; there is a significantly reduced probability of extinction from any population size $S_{t}>1$). This distinction aids our interpretation of which factors contribute to extinction, as the former probability is given particular significance in continuous models. As a disadvantage of this model, parameters should be chosen under the constraint $0\leq f_{0},f_{1},f_{2}\leq 1$, e.g., $l_{i}$ cannot exceed 2. In what follows, we set $f_{0}=0.3$.

## 3 Methods

The simulation method proceeds as follows. At each time step, random numbers are generated in two different manners. First, a selection is made between $l_{1}$ and $l_{2}$, based on the specified probabilities $p_{1}$ and $p_{2}$. Next, a random integer k is generated for each individual according to the model‐specific distribution corresponding to the selected rate, either $l_{1}$ or $l_{2}$. This process is repeated sequentially through each time step across numerous iterations to generate a growth trajectory of the population. The entire simulation is conducted multiple times to obtain an average across possible realizations. Whichever model is used, the geometric mean is uniquely determined by the parameters $l_{1},l_{2}$, and $p_{1}$ (with $p_{2}=1-p_{1}$). We evaluate extinction (or survival) rates numerically by varying these parameters systematically to analyze their relationship with the geometric mean. Simulations were conducted using custom code developed in Mathematica.

**FIGURE 2:**
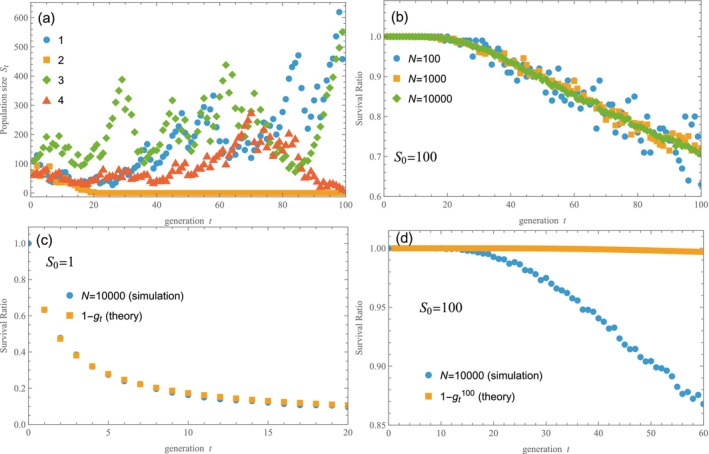
Numerical simulations (Model 1). (a) Four trajectories from $S_{0}=100$ are shown by way of example (N=4). Samples 1 and 3 tend to increase after t=100, while Sample 2 goes extinct ($S_{t}=0$) at t=21 and Sample 4 is about to go extinct at t=100. (b) The survival ratio for $S_{0}=100$, evaluated with the total number of simulations N=100, 1000 and 10,000. (c) The survival ratio for $S_{0}=1$. The numerical result obtained with N=10,000 align closely with the theoretical result derived from the generating function $1-g_{t}$. Consequently, both sets of data almost overlap each other. (d) For $S_{0}=100$, the numerical result and the theoretical result $1-g_{t}^{100}$ deviate from each other.

Let us explain how we evaluate the main results presented in the next section. Figure 2a show simulation results for four sample realizations of the random process in Model 1 ($l_{1}=0.76$, $l_{2}=1.16$, $p_{1}=0.33$ and $S_{0}=100$). The growth rate at each time step takes a definite value of either $l_{1}$ or $l_{2}$ randomly with probability of $p_{1}$ or $1-p_{1}$. Thus, the growth rates at all time steps vary for each simulation run (realization). The first and third realizations (trajectories) tend to grow up at the right end (t=100). The fourth one is decaying and the second goes extinct at t=21. Therefore, the survival ratio at t=100 is three in four, or 0.75. As we increase the number of realizations, N, the survival ratio converges to a certain number. Figure 2b shows the survival ratio evaluated for *N* = 100, 1000 and 10,000, indicating convergence toward about 0.75 at t=100. While a sample size of N∼10,000 is enough to provide meaningful insights, significantly larger sizes are required to reveal detailed features (as in Figure 4).

We are interested in the survival ratio, but not in the size of survived populations. The survival ratio exhibits a notable dependence on the initial population size. In Figure 2c,d, the survival ratios for the initial population size $S_{0}=1$ and $S_{0}=100$ are shown, respectively. For comparison, the theoretical results obtained with a generating function, $1-g_{t}^{S_{0}}$, are also plotted (Appendix A). For a small population, the numerical result agrees with the theoretical result (Figure 2c). However, this is not the case for a large population (Figure 2d). The discrepancy between simulation and theory (Figure 2d) arises because individuals do not have independent lines of descent as they share a common history of random environments. Indeed, the survivability of a population is significantly enhanced if individuals are dispersed to different places to experience different histories. The discrepancy vanishes in the special case of each individual having its own history (Figure 2c). Incidentally, the theoretical result, $g_{t}$, is easy to evaluate for Model 2, which helps us for checking purposes. In what follows, we evaluate the survival ratio for a population with $S_{0}=100$.

## 4 Results

### 4.1 Case 1: *E* = const.

We present the results for cases under specified constraints. In the first case, we vary the growth rates, $l_{1}$ and $l_{2}$, and the probability, $p_{1}$, such that the mean growth rate E is held constant. In terms of $l_{1}$ and $p_{1}$, the parameter $l_{2}$ is given by

(7) $$l_{2}=\left(E-p_{1}l_{1}\right)/\left(1-p_{1}\right).$$

**FIGURE 3:**
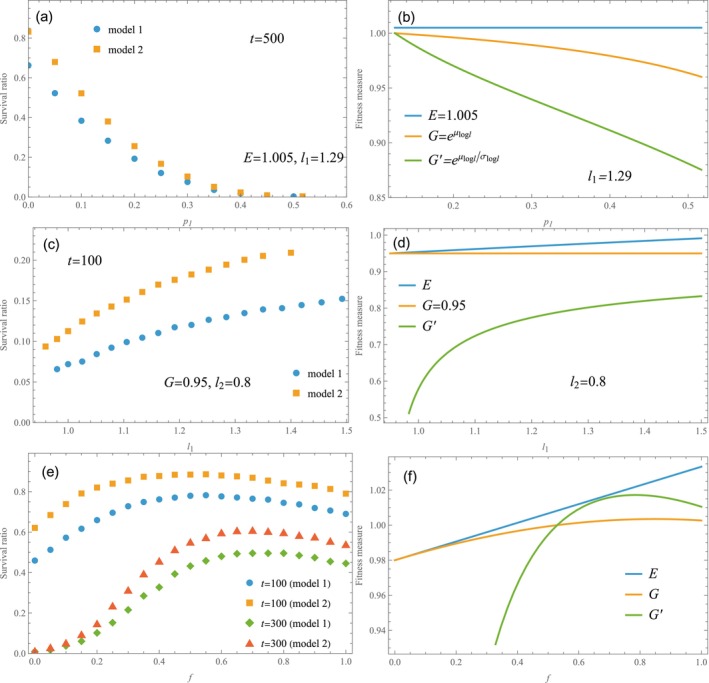
Survival ratio (a, c, e) and fitness measures (b, d, f). (a, b) Survival ratio and E, G and $G^{\prime }$ are plotted against the probability $p_{1}$. The arithmetic mean E and the growth rate $l_{1}$ are held constant at 1.005 and 1.29. The growth rate $l_{2}$ is given by Equation (7). (c, d) Survival ratio and E, G and $G^{\prime }$ are plotted against $l_{1}$, where the geometric mean G and $l_{2}$ are held constant at 0.95 and 0.8. The probability $p_{1}$ is given by Equation (8). (e, f) Survival ratio and E, G and $G^{\prime }$ are plotted against the parameter f in Equation (9) (N=10,000 and $S_{0}=100$).

In Figure 3a, the survival ratio evaluated as explained above is plotted against $p_{1}$ for E=1.005 and $l_{1}=1.29$ (N=10,000). In both models, the survival ratio decreases as $p_{1}$ increases. As $p_{1}$ increases, $l_{2}$ decreases, and geometric mean G decreases. For comparison, G and $G^{\prime }$ are plotted against $p_{1}$ in Figure 3b.

As seen in Figure 3a,b, the survival ratio varies like G and $G^{\prime }$, while E is held constant. The survival ratio decreases with $p_{1}$ because the environmental variance increases with $p_{1}$. Keeping a constant value of $l_{1}$ above the mean growth rate ($l_{1}=1.29,E=1.005$) means that $l_{2}$ decreases as environment 1 becomes more common, which consequently increases the variance in growth rates. In almost all cases, population size blows up exponentially for E>1 while the population goes extinct almost certainly for $p_{1}>0.5$ (Figure 3a). This is because some exceptional realizations that avoid extinction make significant contribution to the mean population size, despite their number being statistically insignificant.

### 4.2 Case 2: *G* = const.

In the second case, we vary parameters while holding the geometric mean G constant. In terms of $l_{1}$ and $l_{2}$, the probability $p_{1}$ is given by

(8) $$p_{1}=\frac{\mu_{\log l}-\log l_{2}}{\log l_{1}-\log l_{2}},$$

where $\mu_{\log l}$ is defined in (4). We vary the parameter $l_{1}$ while keeping $l_{2}$ as constant. For G=0.95 and $l_{2}=0.80$, Figure 3c shows the survival ratio at t=100 (N=10,000). The survival ratio approaches zero in the limit t→∞ for G<1. In Figure 3d, E, G and $G^{\prime }$ are plotted against $l_{1}$. Contrary to expectation, the survival ratio is not constant (Figure 3c). Change in the survival ratio is in line with the prediction of the new measure, $G^{\prime }$ (Figure 3d). In this case, $p_{1}$ decreases as $l_{1}$ increases (Equation 8). As $l_{1}$ deviates from $l_{2}=0.80$, $G^{\prime }$ increases because $\sigma_{\log l}$ increases while $\mu_{\log l}$ remains constant and negative. The results indicate that a larger $\sigma_{\log l}$ corresponds to a larger survival ratio at t=100. In other words, the survival ratio tends to approach zero more gradually as $\sigma_{\log l}$ (or $G^{\prime }$) increases. These findings are corroborated by the results detailed at the end of the next subsection.

### 4.3 Case 3: Applications

We revisit two specific examples from existing literature where geometric‐mean fitness has been utilized (Philippi and Seger 1989; Jansen and Yoshimura 1998). The growth rates, $l_{1}$ and $l_{2}$, depend on a parameter f, which is regarded as a numerical representation of a population's strategy. In our simulations, the parameter f is treated as a constant and no random numbers are generated for it.

In the first example, we consider

(9) $$l_{1}=0.7f+0.98\left(1-f\right),\;l_{2}=1.2f+0.98\left(1-f\right),$$

with $p_{1}=1/3$ and $p_{2}=2/3$ (Jansen and Yoshimura 1998). This model describes individuals allocating their offspring between two distinct habitat types in proportions f and 1−f. Habitat 1 offers high quality but is subject to occasional catastrophes with probability p. In contrast, Habitat 2 provides a stable environment without risk of such events, though at lower quality. Assuming f is genetically determined, the optimal genotype f is selected to maximize geometric‐mean fitness (Yoshimura and Jansen 1996; Jansen and Yoshimura 1998).

At f=1 (all in Habitat 1), the growth rate fluctuates between 0.7 and 1.2 with probability 1/3 and 2/3, respectively. At f=0 (all in Habitat 2), we have $l_{1}=l_{2}=0.98$ $\left(<1\right)$, i.e., a steady decrease of population. The arithmetic‐mean growth rate E increases linearly with f (from 0.98 to 1.03), while the geometric‐mean G has a broad peak around f≃0.8 (Figure 3f). Our simulation results for the survival ratio are presented in Figure 3e (N=10,000). For comparison, $G^{\prime }$ is also plotted in Figure 3e.

The results in Figure 3e,f illustrate an optimal allocation of reproductive effort (Jansen and Yoshimura 1998). Owing to the linear dependence of E on f, arithmetic mean offspring number is maximized at an endpoint, f=1 (all offspring in Habitat 1). Contrary to this conclusion, long‐term survival favors an intermediate value (0<f<1), representing a bet‐hedging strategy that diversifies offspring across two habitats (Yasui 2022). Indeed, G and $G^{\prime }$ in Figure 3f exhibit peaks at f≃0.85 and f≃0.8, respectively. The numerical simulations in Figure 3e indicate similar results in favor of diversification. Therefore, these findings align with previous research that highlights the long‐term benefits of a bet‐hedging strategy based on maximizing geometric‐mean fitness (Cohen 1966; Seger and Brockmann 1987; Philippi and Seger 1989; Yoshimura and Clark 1991). It is noted, however, that the optimal proportion f that maximizes the survival ratio (Figure 3e) depends on the generation number t to be referenced. If the geometric‐mean growth rate is less than unity, every population goes extinct ultimately, i.e., the survival ratio goes to zero with probability one. Therefore, the survival ratio is used as a relative measure with which to compare the long‐term fate of two strategies competing with each other. Nonetheless, an optimal strategy need be addressed on a case‐by‐case basis, because it may depend on specific details of the ecological system.

In the second example, we consider

(10) $$l_{1}=0.6f+1.0\left(1-f\right),\;l_{2}=1.0f+0.58\left(1-f\right),$$

and $p_{1}=p_{2}=1/2$. This model describes various strategies in a unified manner. Assuming that good and bad years occur randomly with equal frequency, phenotypes A (f=0) and B (f=1) are good and bad‐year specialists, while D (f=1/2) is a bet‐hedger that expresses equal proportions of the A and B phenotypes. It is argued that the bet‐hedge strategy D would be evolutionarily stable against A and B because it has the highest geometric‐mean fitness (Philippi and Seger 1989). There are conservative and diversified bet‐hedging strategies. Conservative bet hedging decreases fitness variance while diversified bet hedging increases it, both at the cost of reducing expected fitness (E). Consequently, strategies D and A exemplify conservative and diversified bet hedging, respectively, compared to strategy B, which achieves the highest mean fitness (E=0.795).

**FIGURE 4:**
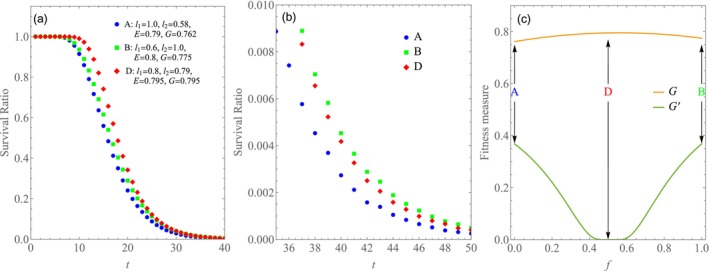
Reversal of optimal strategy. Survival ratios are plotted against the generation number t. Specialist strategies A (f=0) and B (f=1) are compared with a generalist strategy D (f=1/2) for $l_{1}$ and $l_{2}$ in Equation (10), Model 1, *N* = 1,000,000. (a) Strategy D has a larger survival ratio than A and B initially. (b) Strategy B outcompetes D eventually. Indeed, the survival ratio of D appears to become the smallest ultimately (not shown). (c) Strategy D has a larger value of geometric mean G than A and B, while $G^{\prime }$ is larger for B than for D. $G^{\prime }$ is suppressed around f≃0.5 because $\sigma_{\log l}$ becomes almost zero (no difference between $l_{1}$ and $l_{2}$).

Figure 4a,b show the survival ratios for the three cases in Model 1. An enlarged view of the tail of Figure 4a is presented in Figure 4b. A simulation size of N=1,000,000 was required to confirm these details. In Figure 4c, G and $G^{\prime }$ are plotted against f. Figure 4a shows that strategy D initially exhibits a higher survival ratio than strategies A and B (t<30). However, the order of B and D reverses in the long term (t>40), as illustrated in Figure 4b. This pattern is consistent with the trends observed in Figure 4c for G and $G^{\prime }$. Strategy B has a larger value of $G^{\prime }$ than strategy D. The differences observed between A and B in Figure 4a,b are attributable to a small difference in their value of G. Although not shown in the figure, the survival ratio of D appears to become the smallest ultimately. Consequently, our numerical results challenge the original argument by demonstrating that this example does not support a long‐term advantage for bet‐hedging strategies (D and A) compared to strategy B, which has the highest arithmetic‐mean fitness. Although the differences between these strategies are extremely small. These numerical findings present a cautionary note for bet‐hedging strategies that rely solely on long‐term measures. They suggest that the relative order of long‐term survivability could be influenced by the specific duration or term length considered, challenging assumptions about consistent outcomes across different time frames.

**FIGURE 5:**
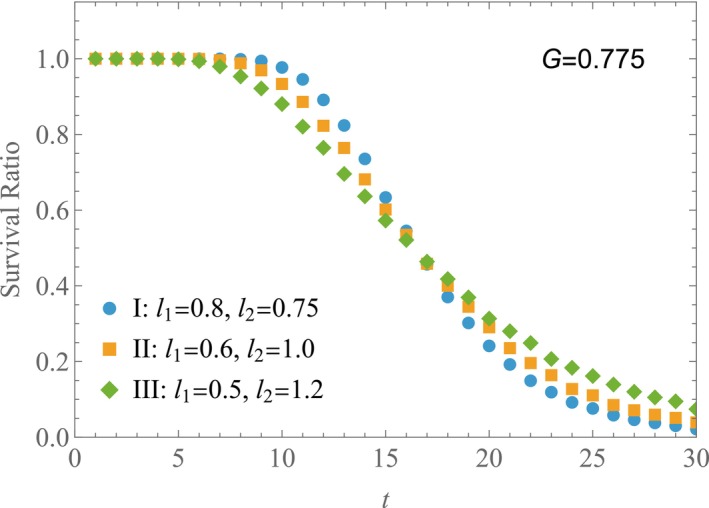
Variation in survival ratio with fixed geometric‐mean fitness. Survival ratios are shown for three cases. Case I: $l_{1}=0.8,l_{2}=0.75$. Case II: $l_{1}=0.6,l_{2}=1.0$. Case III: $l_{1}=0.5,l_{2}=1.2$. $p_{1}=p_{2}=0.5$, $S_{0}=100$, and N=10,000. All cases have a common value of G=0.775, while $G^{\prime }=0.00$, 0.37 and 0.56 for I, II and III, respectively.

Finally, we explore how survival ratios differ among three cases that share a common geometric‐mean fitness value. Figure 5 displays the survival ratios for Cases I, II and III. In Case I, the values for $l_{1}$ and $l_{2}$ are 0.8 and 0.75, respectively. For Case II, these values are 0.6 and 1.0, while in Case III, they are 0.5 and 1.2. The simulations were conducted with parameters $p_{1}=p_{2}=0.5$, $S_{0}=100$, and N=10,000. All cases maintain a geometric‐mean fitness value of G=0.775, while differing in their values of $\sigma_{\log l}^{2}$ and $G^{\prime }$; specifically, $G^{\prime }=$0.00, 0.37, 0.56 for Cases I, II and III, respectively. As shown in Figure 5, the survival ratio for Case III exceeds that of the other two cases when t>15. Notably, as $G^{\prime }$ increases, the tail of the survival ratio curve becomes more pronounced. This dependence on $\sigma_{\log l}^{2}$ and $G^{\prime }$ contributes to the observed reversal in the survival ratio shown in Figure 4. While keeping $l_{1}$ constant, a slight increase in $l_{2}$ from Case I leads to a minor rise in G (Case I′). Although Case I′ has a higher G value than Case III, it may still exhibit lower survival ratio values than Case III. This indicates a reversal in the relationship between geometric‐mean fitness G and survival ratio.

## 5 Discussion

A recent study indicates that geometric‐mean fitness alone does not sufficiently predict when bet hedging becomes adaptive (Weissman et al. 2025). According to this study, the probability of fixation inverts for a population size $S_{0}≃10^{2}$ when the difference in geometric‐mean fitness is approximately 0.01. Our results align with this conclusion by demonstrating that the variance of the logarithmic growth rate and the differences in model assumptions play a significant role in determining the numerical degree of effects, as shown in Figures 3 and 4. The model dependence deserves special mention. If we compare two models in Figure 3c,d, the survival probability comes out to be smaller for Model 1 than for Model 2 due to different modeling assumptions. In practice, the growth rate under a certain condition is evaluated as the conditional expectation of empirically counted numbers. As a stochastic variable, the growth rate randomly takes one of several potential values. Each value is associated with an empirical average observed under particular environmental conditions. However, it is often impossible to gather enough information to confirm the probability distribution of sample values, while the average can still be reliably obtained. To give an example, we obtain the growth rate of 1.2 when five individuals give rise to six individuals. The birth process may be due to one individual bearing two and the other four one $\left(2+4\times 1\right)$, or maybe one bears six and the others none $\left(6+4\times 0\right)$. The average value (of 6/5=1.2) provides limited insight into how offspring are distributed among parents. Among several factors, the probability that an individual will not produce any offspring plays a crucial role in determining whether a population goes extinction or not. This is the reason why we considered two models of the birth process. The Poisson distribution (in Model 1) is commonly assumed in mathematical approaches because of ease in analytical handling. Indeed, the importance of considering other distributions has been investigated (Fox and Kendall 2002; Lloyd‐Smith et al. 2005; Melbourne and Hastings 2008). Thus, the model dependence as we showed in Figure 3 may have an impact on quantitative details in the long‐term tail of survived populations, especially when we are interested in an optimal strategy in the long term.

The use of geometric‐mean fitness has been recently criticized in other contexts than bet‐hedging. While the mean growth rate is widely used as a fitness measure, the importance of its variance has also been acknowledged in recent studies (Tuljapurkar and Orzack 1980; Tuljapurkar et al. 2009; Trotter et al. 2013; Zhou and Xue 2022; Dinis et al. 2022; Cavallero et al. 2025). In modern coexistence theory, the special focus is placed on invasibililty, namely the probability of a single invader to establish (Chesson 2003; Barabás et al. 2018; Chesson 2018). While the mean growth rate is considered a good measure of invasibility (Chesson 2003), it does not agree with how the probability of invasion varies (Pande et al. 2020). Although not obvious, it is plausible that the measure of invasibility is somewhat related to the measure of long‐term persistence with which to rationalize bet‐hedging strategies. Since bet‐hedging theories argue for the long‐term benefit of a bet‐hedging strategy without any recourse to competition (Cohen 1966; Cooper and Kaplan 1982; Seger and Brockmann 1987; Philippi and Seger 1989; Yoshimura and Clark 1991; Haaland et al. 2019), we dealt with population growth models with variable growth rates without assuming intraspecific competition.

Empirical studies have suggested that bet‐hedging mechanisms operate in a wide range of plant and animal species, as rationalized by “the geometric‐mean principle” (Seger and Brockmann 1987). Animals and plants may evolve adaptations to avoid extinction, namely bet‐hedging traits for the benefit of long‐term persistence (Seger and Brockmann 1987; Philippi and Seger 1989). To name a few, seed dormancy (Cohen 1966), insect diapause (Joschinski and Bonte 2021), foraging (Burns and Dyer 2008), oviposition (McLaughlin and Wasserberg 2021), multiple mating (Yasui 2001), reduced reproductive output (Lovich et al. 2015), etc. In the past, geometric‐mean fitness has served as a long‐term measure (Yoshimura and Clark 1991; Yoshimura and Jansen 1996), ever since introduced to account for seed dormancy (Cohen 1966). Revisiting this guiding principle, the present study demonstrates that the geometric mean does not precisely correspond to the survival ratio (Figure 3c,d), although it can still serve as a useful guideline. The present findings suggest us to reconsider reported examples of biological bet‐hedging carefully. Delayed germination in desert annuals is the classic example addressed by Cohen (1966). Producing a fraction of dormant seeds acts as the hedge against unpredictable variation in reproductive success. Cohen's model determines what proportion f of the seed production should be germinated, provided the probability p of the year to be good for reproduction, i.e., the fraction of seeds that germinate should be correlated with rainfall. Indeed, this prediction has been positively tested by empirical studies (Philippi and Seger 1989). A similar correlation may be shown for yearly variation in reproductive success (Venable 2007).

The present study focused on the validity of the long‐term fitness concept, especially regarding the application to evolutionary bet hedging theory, while leaving out various ecological factors. Among others, the density dependence would be the most important that may affect the long‐term fate of populations with competing genotypes (Hassell et al. 1991; Hilborn 1975; Keeling 2000). The crucial issue of bet hedging lies in that the genotype expressing bet‐hedging phenotypes may be favored over alternatives with larger expected offspring numbers. In other words, bet hedging is the preference of long‐term benefit over short‐term benefit. As a matter of fact, it is not at all trivial not only whether but under what conditions it holds true. We expect that some measure like geometric mean G remain valid, at least approximately, as a relative indicator of long‐term persistence, even in the presence of a finite carrying capacity. In the case where E<1 and G<1, as in Figure 4, any population goes extinct eventually, regardless of strategy. Therefore, the density effect would be of minor importance while the strategy with a large G value outperforms other strategies in early stages (Figure 4a). In cases where E>1 and G<1, populations that tend to grow exponentially will be impacted by a finite carrying capacity, although such cases are expected to be rare. Typical populations' persistence should conform to expectations based on long‐term measures. Specifically, those populations exhibiting the highest values in these measures are likely to outcompete others. In the case G>1, not covered in this study, the population can go extinct in the presence of carrying capacity but not without it. It is not obvious whether a long‐term measure plays a significant role under such a case. Like the two models in the present study, we may think of many ways to take account of the density effect consistently with demographic stochasticity, e.g., through a suppressive effect on the growth rate, by suppressing the maximum offspring number (as we did in Model 2, Figure 1), and/or through the death process while treating birth and death processes separately. To see if the density effect has a significance is left as a future work.

## Author Contributions

**Takuya Okabe:** conceptualization (lead), formal analysis (lead), funding acquisition (equal), investigation (lead), methodology (lead), project administration (lead), software (lead), visualization (lead), writing – original draft (lead), writing – review and editing (lead). **Jin Yoshimura:** conceptualization (supporting), writing – original draft (supporting), writing – review and editing (supporting). **Hiromu Ito:** funding acquisition (equal), visualization (equal), writing – original draft (supporting), writing – review and editing (supporting).

## Funding

This work was supported by Japan Society for the Promotion of Science (23KK0210, 25K01465, 21K12047, 25K22145).

## Conflicts of Interest

The authors declare no conflicts of interest.

## Data Availability

The datasets analyzed during the current study are generated with the code available from the Dryad repository: https://doi.org/10.5061/dryad.0k6djhbdq.

## Appendix A

For comparison, we present an analytical approach assuming that individuals thrive independently of one another. As noted in the Models section, let $f_{k}$ represent the probability that an individual has k offspring. Denote as $g_{t}$ the probability that a single individual in generation t has no offspring. The relationship between $g_{t+1}$ and $g_{t}$ can be expressed by the following equation:

$$g_{t+1}=\sum_{k}f_{k}{\left(g_{t}\right)}^{k}.$$

Here, an individual produces k offspring with probability $f_{k}$, and the probability that all k individuals have no offspring is ${\left(g_{t}\right)}^{k}$. This equation sums over all possible numbers of offspring to determine the extinction probability at generation t+1. The function $g_{t}$ is determined by solving this recurrence relation.

Assuming that $S_{0}$ individuals are independent, their collective extinction probability at generation t can be calculated as $g_{t}$ raised to the power of $S_{0}$, $g_{t}^{S_{0}}$. This probability is illustrated in Figure 2 for comparison purposes. While this calculation assumes independence, in reality, individuals experience common environmental variations.
